# Valorization of Rice Husk (*Oryza sativa* L.) as a Source of In Vitro Antiglycative and Antioxidant Agents

**DOI:** 10.3390/foods12030529

**Published:** 2023-01-25

**Authors:** Ilaria Frosi, Daniela Vallelonga, Raffaella Colombo, Chiara Milanese, Adele Papetti

**Affiliations:** 1 Department of Drug Sciences, University of Pavia, 27100 Pavia, Italy; 2 Department of Chemistry, Physical Chemistry Section, University of Pavia and C.G.S.I., 27100 Pavia, Italy

**Keywords:** rice husk, glycation reaction, antiglycative agents, antioxidant activity, polyphenols

## Abstract

Rice husk is a good source of polyphenols, but it has not been efficiently utilized in food applications yet. Therefore, the aim of this work is to investigate, by in vitro assays, the polyphenolic extract (RHE) capacity of this waste to counteract the protein glycation at different stages of the reaction, correlating this activity with the antiradical properties. A microwave-assisted extraction using hydro-alcoholic solvents was applied to recover husk polyphenols. Extraction parameters were optimized by the design of the experiment. The extract with the highest polyphenolic recovery was obtained at 500 W and 90 °C, using 1:35 g of dry material/mL solvent, 80% ethanol, and a 5 min extraction time. Results highlight the ability of RHE to inhibit the formation of fructosamine in the early stage of glycation with a dose-dependent activity. Furthermore, in the middle stage of the reaction, the highest RHE tested concentration (2.5 mg/mL) almost completely inhibit the monitored advanced glycation end products (AGEs), as well as showing a good trapping ability against α-dicarbonyl intermediates. A strong positive correlation with antioxidant activity is also found. The obtained results are supported by the presence of ten polyphenols detected by RP-HPLC-DAD-ESI-MS^n^, mainly hydroxycinnamic acids and flavonoids, already reported in the literature as antiglycative and antioxidant agents.

## 1. Introduction

Rice (*Oriza sativa* L.) is the third food crop most produced and consumed worldwide, with about 750 million tons per year [1]. Therefore, a high quantity of by-products is obtained from rice cultivation and commercial processing, among which husk represents about 20% of the rice production’s total weight. Nowadays, rice husk (RH) is commonly used in non-food applications such as biofuel production and animal feed [2], but a large amount of it is not efficiently utilized. The main consequence of its underutilization is an environmental problem derived from its burning in the field (loss of soil moisture and air pollution).

Despite this, RH contains high amounts of phenolic acids, in particular vanillic and *p*-coumaric acids, thus showing a high in vitro antioxidant capacity (DPPH radical scavenging activity and ferric reducing antioxidant power), which strictly depends on the growth sites [3,4,5,6,7]. The bound and free polyphenolic content of RH is generally higher than that registered for bran [8]. In the last decade, many different biological properties have been attributed to the RH polyphenolic fraction, i.e., cellular antiproliferative and antioxidant activities [9] and inhibitory activity against different Gram-negative bacterial strains and fungi (*Aspergillus niger* and *Histoplasma capsulatum*) [10]. In addition, an acidified methanolic extract of purple rice husk was shown to possess antimutagenicity against aflatoxin B1-induced mutagenesis in both bacterial and animal models, mainly due to vanillic acid [11]. Interesting antihyperglycemic activities were reported for paddy husk, a by-product produced in the pudding milling industry [12].

Phenolic acids have been reported in literature to be able to inhibit the early stage of the glycation reaction, which is considered the main process involved in the development of many chronic diseases such as type 2 diabetes, nephropathy, osteoporosis, cardiovascular, and neurodegenerative disorders [13]. The glycation is a non-enzymatic and spontaneous reaction that occurs between an amino group of proteins, lipids, and nucleic acids, and the carbonyl group of glucose or other reducing sugars, resulting in the initial formation of Amadori products, such as fructosamine. These instable products can be transformed into dicarbonyl compounds, such as methylglyoxal (MGO) or glyoxal (GO), which undergo different reactions that contribute to the formation of advanced glycation end products (AGEs) [14]. Caffeic, vanillic, ferulic, chlorogenic, and coumaric acids inhibited fructosamine and AGEs formation and the crosslinking of proteins thanks to the formation of H-bonds with amino acid residues in proteins, acting as antiglycative agents [15,16]. Considering the above-mentioned literature results and the fact that the search for novel natural antiglycative compounds is increasing day by day, the aim of the present research work is to recycle RH by extracting the polyphenolic fraction and testing it for its potential antiglycative capacity. A green approach was used to extract polyphenols by applying a microwave-assisted method and using an aqueous-ethanol mixture as solvent, and the extraction conditions were optimized using experimental designs. The obtained extract with the highest polyphenolic recovery (RHE) was evaluated for its antiradical activity against DPPH and ABTS stable radicals and for its capacity to inhibit the glycation reaction at different stages and to directly trap dicarbonyl compounds, well-known intermediate compounds in the AGEs generation.

## 2. Materials and Methods

### 2.1. Reagents

All solvents and reagents (grade purity 97–99%) used were provided by Merck KGaA (Dermstadt, Germany), with the exception of kaempferol-3-*O*-glucoside which was purchased from Extrasynthese (Genay, Rhone, France). Water was obtained from the Millipore Direct-QTM system (Merk-Millipore, Milan, Italy).

### 2.2. Plant Material

Organic white rice (*Oryza sativa* L.) husk was provided by a local organic farm (DiCristiana Azienda Agricola, Robbio, Pavia, Italy). Husk was dried overnight in an oven at 45 °C to a final residual moisture of about 6%, and subsequently, it was minced into powder with a chopping knife and passed through a 500 μm sieve screen. The so-obtained sample was stored at 4–8 °C in amber glass bottles prior to extraction.

### 2.3. Microwave-Assisted Extraction Process

The Microwave-Assisted Extraction (MAE) process was performed using a microwave apparatus equipped with a teflon vessel system (Ethos LEAN, Sorisole, Italy). The temperature was gradually increased in 5 min to the selected extraction temperature (energy power of 800 W maximum), then samples were held at the extraction temperature using a power of 500 W maximum. Briefly, 500 mg of RH powder was accurately weighted and placed into an extraction vessel. The extraction was performed under operating conditions determined by experimental designs, with different solvents for solid material amounts (15–35 mL/g), temperatures (40–90 °C), hydro-alcoholic mixtures (30–80% ethanol), and irradiation times (5–35 min). After a ventilation period of 10 min, each sample was filtered through a paper filter (Cordenons Technical Papers, 90 gsm). Ethanol was evaporated under vacuum (about 145 psi) at 37 °C (Buchi R-II, Buchi, UK), and the remaining aqueous suspension was freeze-dried (Modulyo freeze-dried s/n 5101, Akribis Scientific Limited, Cheshire, UK) to obtain a stable solid product.

### 2.4. Experimental Design

Statgraphics Centurion 19 software (Statgraphics Technologies, Inc. The Plains, VA, USA) was used for experimental design, model fitting, and data analysis.

#### 2.4.1. Screening Study of Process Variables

Preliminary experiments were conducted to determine the effect and the interactions among the operating extraction conditions in the MAE process on the recovery of phenolic compounds from rice husk. A 2^4^ full factorial design was selected to study four independent variables on two levels, including the solvent to solid ratio (X_1_, 15–35 mL/g), temperature (X_2_, 40–80 °C), ethanol percentage (X_3_, 30–70%), and time (X_4_, 5–35 min). A total of 19 experimental runs, including three repetitions for the central point, were generated (Table 1). The considered response variable was the phenolic compound yield expressed as the total area of all the peaks recorded in RP-HPLC-DAD chromatograms at 320 nm (wavelength with a higher intensity signal).

The design matrix for the experiment and the regression model for the response were calculated as follows:

(1) $$Y=\beta_{0}+\overset{n}{\underset{i=1}{\sum }}\beta_{i}X_{i}+\overset{n}{\underset{1\leq i\leq j}{\sum }}\beta_{i}X_{i}X_{j}+\varepsilon$$

where Y is the predicted response (yield); *n* is the number of variables; *X_i_* and *X_j_* are the independent variables; β_0_ defines the fixed response at the central point; β*_i_* and β*_ij_* are the linear, and the interaction coefficients *ε* is a random error. An analysis of variance (ANOVA) was carried out to determine any significant differences (*p* < 0.05) among the applied extraction conditions.

#### 2.4.2. Response Surface Methodology

The response surface methodology (RSM) was employed to optimize the extraction conditions in the MAE process. On the basis of the screening study, two independent variables were further optimized, namely temperature (X_1_, 60–90 °C) and ethanol percentage (X_2_, 70–80%), while maintaining a constant extraction time (5 min) and the solvent to solid ratio (35 mL/g). A three-level Box-Behnken design (BBD) was applied, with 10 experimental runs planned, including two repetitions at the central point (Table 2).

The considered response variable was the phenolic compound yield determined by RP-HPLC-DAD analysis, as reported for the screening design. BBD was a second-order polynomial model with coded independent variables (x_j_, j) that followed the reported equation:

(2) $$Y=\beta_{0}+\overset{n}{\underset{i=1}{\sum }}\beta_{i}X_{i}+\overset{n}{\underset{i=1}{\sum }}\beta_{ii}X_{i}^{2}+\overset{n}{\underset{1\leq i\leq j}{\sum }}\beta_{ij}X_{i}X_{j}+\varepsilon$$

where Y is the predicted response (yield); *n* is the number of variables; *X_i_* and *X_j_* are the independent variables; β_0_ defines the fixed response at the central point; β*_i_*, β*_ii_* and β*_ij_* are the linear, quadratic, and interaction coefficients; and *ε* is a random error. An analysis of variance (ANOVA) was carried out to determine any significant differences (*p* < 0.05) among the applied extraction conditions. The adequacy of the constructed quadratic model was assessed on the coefficient of determination (R^2^), adjusted coefficient (R^2^_adj_), and prediction error sum of squares (PRESS). Three extra tests in duplicate were also carried out to verify the predicted data under the optimal conditions assessed by RSM-BBD.

### 2.5. RP-HPLC-DAD Analyses

An HPLC Agilent 1200 system (Waldbronn, Germany) equipped with a mobile-phase online degasser, a quaternary pump, and a diode array detector (DAD) was used. The separation of the analytes was performed on a Gemini^®^ C18 analytical column (150 × 2.0 mm i.d., 5 μm, Phenomenex, Torrance, CA, USA) at 0.3 mL/min flow (injection volume of 20 μL). A binary mobile phase was used: solvent A (0.1% formic acid in water) and solvent B (methanol), and the separation was performed in gradient: 0–10 min, 10–30% B; 10–20 min, 30–45% B; 20–30 min, 45–55% B; 30–35 min, 55–65% B; 35–40 min, 65–75% B; 40–45 min, 75–85% B; 45–48 min, 85% B, followed by the column reconditioning in 12 min. UV-Vis spectral data of samples (RHEs) were acquired in the 200–700 nm range, and chromatograms were recorded at 270, 320, and 370 nm. The ChemStation software was used for data acquisition and processing.

### 2.6. RP-HPLC-DAD-ESI/MS^n^ Analyses

Phytochemicals in RHE were identified using a Thermo Finnigan Surveyor Plus HPLC connected to a LCQ Advantage Max ion trap spectrometer through an ElectroSpray Ionization (ESI) source (Thermo Fischer Scientific, Waltham, MA, USA). The analyses were performed using the same experimental chromatographic conditions reported in 2.5 section. The ion trap operated in data-dependent, full scan (50–1500 *m*/*z*), and MS^n^ to obtain a fragment ion width of 35% and an isolation width of 3 m/z. Chlorogenic acid and kaempferol-3-O-glucoside (10 ppm in 0.1% formic acid-methanol solution, 50:50, *v*/*v*) were used to set up MS parameters: sheath and auxiliary gas flow: 20 and 10 arbitrary units, respectively; ionization voltage, 5 kv; capillary temperature, 300 °C. The analyses were performed in positive e negative ionization modes. The software used was Thermo Fischer Scientific Excalibur 2.1.

### 2.7. Evaluation of Amadori Products Inhibition

Amadori products were measured as fructosamine generated by glucose (GLU) according to Zhang et al. [17] with slight modifications [18,19]. The capacity of RHEs to inhibit fructosamine formation was calculated as follows:

(3) $$\mathrm{inhibition}\%=\left[1-\left(\frac{{\mathrm{Abs}}_{\mathrm{glycated}\mathrm{system}\left(\mathrm{BSA},\mathrm{GLU},\mathrm{extract}\right)}-{\mathrm{Abs}}_{\mathrm{background}\left(\mathrm{BSA},\mathrm{extract}\right)}}{{\mathrm{Abs}}_{\mathrm{glycated}\mathrm{system}\left(\mathrm{BSA},\mathrm{GLU}\right)}-{\mathrm{Abs}}_{\mathrm{background}\left(\mathrm{BSA}\right)}}\right)\right]\times 100$$

### 2.8. Evaluation of Antiglycative Capacities

The antiglycative capacities of RHE were evaluated by bovine serum albumin-glucose (BSA-GLU) system and the bovine serum albumin-methylglyoxal (BSA-MGO) system, as previously reported [18,19,20]. In all systems, aminoguanidine (AG) was used as a positive control, while the freeze-dried extracts were dissolved in water to obtain 0.5, 1, and 2 mg dry matter/mL reaction mixture. The fluorescence intensity (FI) of the mixtures without sample (RHE), and of RHE dissolved in phosphate buffer were recorded as negative control and background, respectively. Pentosidine-like (λ_exc_ 335 nm; λ_em_ 440 nm) AGEs fluorescence was monitored for GLU and MGO systems and argpyrimidine-like (λ_exc_ 370 nm; λ_em_ 440 nm) AGEs fluorescence for MGO systems (PerkinElmer L550B). The capacity of RHE to inhibit AGEs formation was calculated as follows:

(4) $$\mathrm{inhibition}\%=\left[1-\left(\frac{{\mathrm{FI}}_{\mathrm{glycated}\mathrm{system}\left(\mathrm{BSA},\mathrm{GLU}\mathrm{or}\mathrm{MGO},\mathrm{extract}\right)}-{\mathrm{FI}}_{\mathrm{background}\left(\mathrm{BSA},\mathrm{extract}\right)}}{{\mathrm{FI}}_{\mathrm{glycated}\mathrm{system}\left(\mathrm{BSA},\mathrm{GLU}\mathrm{or}\mathrm{MGO}\right)}-{\mathrm{FI}}_{\mathrm{background}\left(\mathrm{BSA}\right)}}\right)\right]\times 100$$

### 2.9. Evaluation of Methylglyoxal and Glyoxal Trapping Capacities

To monitor GO and MGO trapping capacities, the method proposed by Maietta et al. [19] and Mesías et al. [20] was applied using the same experimental chromatographic conditions. RH aqueous solution (0.5, 1, and 2.5 mg dry matter/mL) was used as a sample, while PBS as control sample. GO and MGO trapped percentages were calculated as follows:

(5) $$\mathrm{GO}\mathrm{or}\mathrm{MGO}\mathrm{decrease}\left(\%\right)=\left(\frac{{\mathrm{GO}\mathrm{or}\mathrm{MGO}}_{\mathrm{control}\mathrm{sample}}-{\mathrm{GO}\mathrm{or}\mathrm{MGO}}_{\mathrm{sample}}}{{\mathrm{GO}\mathrm{or}\mathrm{MGO}}_{\mathrm{control}\mathrm{sample}}}\right)\times 100$$

### 2.10. Determination of Free Amino Groups

The *o*-phthaldialdehyde (OPA) assay [21] was used to determine the unreacted lysine groups of glycated materials in the incubated sample. A 1 mL aliquot of OPA reagent (daily prepared) was mixed with a 50 μL aliquot of sample (BSA-GLU system glycated material containing the extract in a final concentration of 0.5, 1, and 2.5 mg/mL), diluted 1:2 with PBS in a 1 mL cuvette. The mixed solution was incubated for 2 min at room temperature, and the absorbance was read at 340 nm. against a blank containing the OPA reagent and untreated BSA as a control (100% free amino groups).

(6) $$\mathrm{Free}\mathrm{lysine}\left(\%\right)=\left(\frac{{\mathrm{Abs}}_{\mathrm{control}}-{\mathrm{Abs}}_{\mathrm{sample}}}{{\mathrm{Abs}}_{\mathrm{control}}}\right)\times 100$$

### 2.11. DPPH Assay

Antiradical activity against the stable-colored DPPH free radical was evaluated as reaction mixture discoloration at 515 nm after 20 min of reaction (Spectophotometer Perkin-Elmer Lambda25) [19]. RHE was tested at 0.5, 1, 2.5, and 5 mg dry matter/mL. The antiradical activity was calculated as follows:

(7) $$\mathrm{Anti}-\mathrm{DPPH}\mathrm{activity}\left(\%\right)=\left(\frac{{\mathrm{Abs}}_{\mathrm{control}}-{\mathrm{Abs}}_{\mathrm{sample}}}{{\mathrm{Abs}}_{\mathrm{control}}}\right)\times 100$$

### 2.12. ABTS Assay

Antiradical activity against the ABTS cation radical (ABTS^•+^) was evaluated according to the method previously described by Re et al. [22]. A 20 μL aliquot of extract (0.5, 1, 2.5, and 5 mg dry matter/mL) or ethanol was used as a sample or control, respectively. RHE’s capacity to scavenge ABTS^•+^ was calculated as follows:

(8) $${\mathrm{ABTS}}^{•+}\mathrm{inhibition}\left(\%\right)=\left(\frac{{\mathrm{Abs}}_{\mathrm{control}}-{\mathrm{Abs}}_{\mathrm{sample}}}{{\mathrm{Abs}}_{\mathrm{control}}}\right)\times 100$$

### 2.13. Statistial Analysis

Results were expressed as the mean ± standard deviations (SD) of measurements obtained from at least three replicated experiments performed in duplicate. Differences were considered significant at *p* < 0.05 and *p* < 0.01. Statistical analysis was carried out using Microsoft Office 365.

## 3. Results and Discussion

### 3.1. Microwave-Assisted Extraction (MAE) Optimization

In this experimental work, to select the optimal extraction conditions, a screening phase followed by an optimization phase was performed. In fact, a full factorial design was first used to identify the significant factors affecting the quality process, and then the response surface methodology was applied to endorse the setting of the process and set the best conditions. Finally, the best conditions were validated by verifying that the experimental phenolic compound yield was in accordance with the predicted value.

#### 3.1.1. Screening Phase

**Figure 1:**
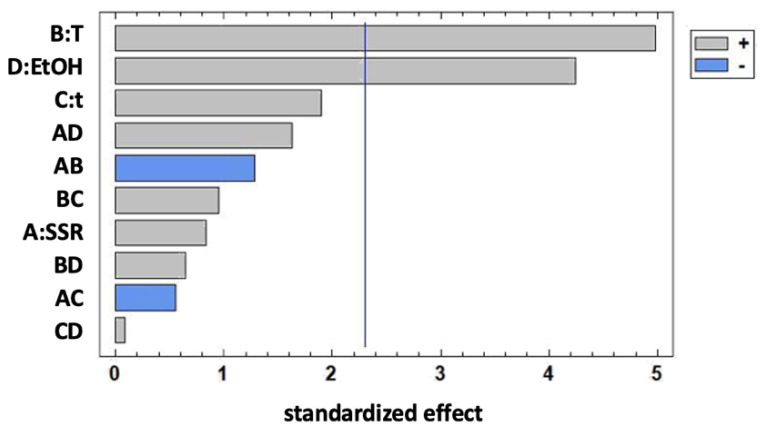
Standardized Pareto chart effect for TMC yield. Gray and blue colors represent the positive and negative effects of the factors on the response variable, respectively. The significance of the effects at a 95% confidence level is tested by the vertical line.

The experimental total metabolite content (TMC) yield values (expressed as the total area of the peaks registered at 320 nm, selected as the λmax of hydroxycinnamic acids) obtained in the screening phase are shown in Table 1. The standardized Pareto chart diagram (Figure 1) highlights the importance and statistical significance of the effects (linear, quadratic, and interaction between variables) of the variables studied in the model. Horizontal bars represented the positive and negative (gray and blue, respectively) effects of the factors on the response variable, and the vertical line tested the significance of the effects at a 95% confidence level.

All the considered variables had a positive effect on the response, but only temperature (T) and the ethanol percentage (EtOH) present in the extraction mixture had a significant effect on the yield. Temperature has a positive effect since it promotes vegetable tissue softening, and weakening of phenol-protein and phenol-polysaccharide interactions. This may favor the polyphenol diffusion into the solvent [23]. Polyphenols are thermolabile compounds that can undergo thermal degradation when exposed to temperatures above 70 °C during conventional extractions [23,24]. However, an in-depth study performed by Liazid et al. [25] pointed out the greater thermal stability of polyphenols when submitted to MAE. In fact, 22 chemically different phenolic compounds were stable up to 100 °C for 20 min, at 500 W. Therefore, in the present study, the temperature range of 60–90 °C was considered for further experiments, and the factors that were not significant in the screening phase were kept constant. In the investigated experimental domain, the hydro-alcoholic mixture containing 70% ethanol provided the highest yields, especially when correlated with high values of the solvent to solid ratio (SSR). In MAE, polar solvents with high dielectric properties are more efficient because of their higher energy absorption and heating generation. Ethanol is considered a stronger energy adsorbent compared to water, and generally, hydro-alcoholic mixtures with a high percentage of ethanol are expected to recover a higher polyphenol yield [26,27]. Our results were in agreement with these observations. The selected time was 5 min, as the application of microwaves generally provides the highest extraction yield within a few minutes and a longer time is not necessary [28,29]. Moreover, SSR was fixed at the highest tested level o(35 mL/g) due to the positive correlation with ethanol percentage.

#### 3.1.2. Optimization Phase

A Box-Behnken design (BBD) approach was used to optimize phenolic compounds MAE from rice husk. Table 2 reported the detailed experimental design along with the experimental and predicted values of extraction yield. The second-order polynomial equation of the obtained response surface was as follows:

Y = 1.08526 × 10^6^ − 20973.3 EtOH − 8219.48 T + 133.341 EtOH^2^ + 36.3732 EtOH T + 43.8095 T^2^ (9)

ANOVA results indicated that both T and ethanol concentrations significantly affected the yield, as well as the interaction between the two considered factors (*p* < 0.05). R^2^ and R^2^_adj_ values of 97.72% and 94.87%, respectively, indicated a close agreement between experimental and predictive values (Table 3).

**Figure 2:**
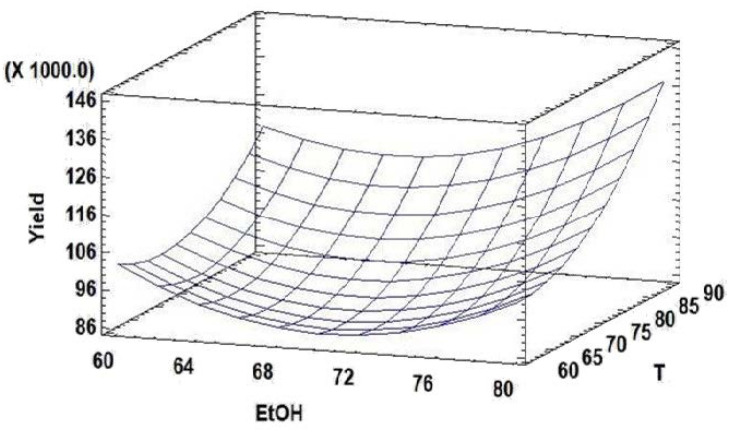
Response surface plots showing the significant effect of temperature (T) and ethanol (EtOH) percentage on TPC yield (expressed as sum of all peaks area, measured in mAu*s).

By analyzing the surface plots obtained for TMC yield as a function of T and ethanol percentage, the optimum values for the two parameters resulting in the highest extraction yield could be found around the higher-level values in the experimental domain (Figure 2). In fact, the statistical software defined 90 °C and 80% ethanol to maximize the extraction yield and calculated 137,771 mAu*s of total peak area with an inferior and superior 95% confidence level for the mean values of 129,139 mAu*s and 146,404 mAu*s, respectively, ranges in which the experimental value must be included.

In order to validate the adequacy of the second-order polynomial equation model, three additional experiments were performed under the optimal predicted conditions: 80% ethanol, 90 °C, 5 min, and 35 mL/g SSR. The obtained extracts were analyzed by RP-HPLC-DAD, and the mean total area of the peaks recorded in the chromatographic profiles at 320 nm was 13,5852.3 ± 1989.8 mAu*s, confirming the adequacy of the fitted model. RHE obtained using the above-reported experimental conditions was selected for further characterization and bioactivity assays.

### 3.2. Identification of Phytochemicals

**Figure 3:**
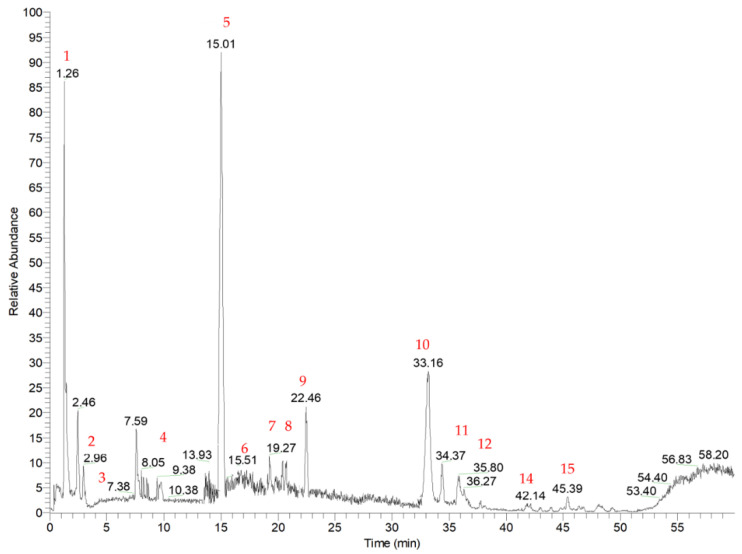
RHE base peak chromatogram registered in negative ionization mode obtained by reverse phase high-performance liquid chromatography coupled to electrospray ionization mass spectrometry (RP-HPLC-DAD-ESI-MS^n^).

The qualitative composition profile of the RHE was obtained by RP-HPLC-DAD-ESI/MS^n^ in data-dependent acquisition mode. The typical base peak chromatogram registered in negative ionization mode is shown in Figure 3. Based on the molecular ions’ mass, retention time, and MS fragmentation patterns, and by considering data reported in the literature, 15 compounds were putatively identified, which belong to different chemical classes, and are listed in Table 4. In RHE, two hydroxycinnamic acids were detected (compounds **1** and **5**). Compound **1** was putatively identified as 1-caffeoyl-β-D-glucose (MM 342). It was present in the MS spectrum as a formic acid adduct (*m*/*z* 387). The loss of the glucosyl moiety (162 Da) resulted in the generation of an ion at *m*/*z* 179, corresponding to caffeic acid, which subsequently lost a water molecule and a carboxylic group, leading to the formation of secondary ions at *m*/*z* 161 and 135, respectively [30]. Compound **5** exhibited a molecular ion at *m*/*z* 163, which fragmented to *m*/*z* 119 [M-H-CO_2_]^−^ [31], and it was identified as *p*-coumaric acid, which was already found in high concentrations in rice husk extracts [4]. Other two small organic molecules, compounds **3** and **4,** were putatively identified as quinic acid (*m*/*z* 191) and vanillin (*m*/*z* 151), respectively. For quinic acid, the loss of water led to the formation of a parent ion at *m*/*z* 173. The subsequent loss of carbon dioxide (44 Da) and a water molecule (18 Da) generated the fragment at *m*/*z* 111 [32]. For compound **4**, the loss of a methyl moiety (15 Da) gave the base peak at *m*/*z* 136, and it was putatively assigned as vanillin, as previously reported by Lopez-Fernandez et al. [31].

Considering flavonoids, compounds **6** and **7** are isobaric and present a molecular ion at *m*/*z* 563 [M–H]^−^. They showed the typical fragmentation pattern of C-glycosyl flavones, at *m*/*z* 383 [(M–H)–120–60]^−^ and 353 [(M–H)–120–90]^−^ corresponding to the addition of 83 amu and 113 amu to the aglycon, respectively; in addition, in the MS/MS spectra, fragment ions at *m*/*z* 473 [(M–H)–90]^−^ and 443 [(M–H)–120]^−^ were present, characteristic of the neutral loss of a glucosyl and pentosyl residues, respectively. These results suggest that compounds **6** and **7** were isomers with an aglycone consisting of apigenin with a pentosyl and a glucosyl moiety linked to positions 6 and 8, respectively. According to different reports on fragmentation of 6,8-di-C-glycosylflavones [33,34,35], preferential cleavage of the sugar moiety occurs at the 6-C rather than the 8-C position. Therefore, compound **6** was proposed to be apigenin 8-C-arabinoside-6-C-glucoside, while compound **7** was proposed to be apigenin 6-C-arabinoside-8-C-glucoside. Compound **10** was putatively identified as tricin by comparing the fragmentation patterns with literature data [36]; it was already identified in rice and other cereal cultivars [37], and it showed an [M–H]^−^ ion at *m*/*z* 329, which was subjected to two subsequent demethylations, giving a base peak at *m*/*z* 314 and a secondary peak at *m*/*z* 299. Compound **12** was putatively assigned to a tricin derivative as it showed the deprotonated aglycone fragment at 329 *m*/z with high intensity and a second fragment at *m*/*z* 314, for the characteristic loss of the methyl moiety [38,39]. In fact, several studies reported the presence of tricin glucosides and lignan derivatives in the leaves and bran of *Oryza Sativa* L. [37,40]. Compound **13** was tentatively identified as 2,7-dihydroxy-4,5-dimehoxyisoflavone, which gave the [M–H]^+^ at *m*/*z* 315 in positive ionization [41]. Among hydroxybenzoic acid derivatives, compound **8** showed a molecular ion at *m*/*z* 187, which fragmented, giving an ion at *m*/*z* 125 by the loss of carbon dioxide (44 Da) and one molecule of water (18 Da), and *m*/*z* 169 by the loss of a molecule of water (18 Da): it was tentatively identified as hydroxygallic acid, as it has a similar fragmentation pattern to gallic acid pattern [42,43]. Considering fatty acids, compounds **2** and **15,** showing molecular ions at *m*/*z* 133 and *m*/*z* 295, were identified as levulinic acid and hydroxy-octodienoic acid, respectively. Levulinic acid was already found in rice husk, and the proposed fragmentation is due to the loss of water (18 Da) and carbon dioxide (44 Da) from the molecular ion [M–H]^−^ at *m*/*z* 133, giving two daughter ions at *m*/*z* 115 and *m*/*z* 71 [44]. Compound **15** presented the fragment ions at *m*/*z* 277 and *m*/*z* 171 generated from a neutral loss of water and the cleavage of the C-C bond adjacent to the hydroxyl group, respectively [45]. Compound **11** was tentatively assigned to a hydroxy oleic acid derivative, by comparing the fragmentation patterns of fatty acids previously identified in rice protein isolate extracts [46]. It showed a molecular ion at *m*/*z* 329, which gave a base peak ion at *m*/*z* 229, corresponding to the carboxylate anion of the oleic acid, and provided secondary fragment ions at *m*/*z* 314 [M–H–CH_3_]^−^, *m*/*z* 311 [M–H–H_2_O]^−^, *m*/z 211, and *m*/*z* 171.

**Table 4:** HPLC-MS and MS^n^ data of the identified compounds in RHE. Compounds are reported in order of elution; * positive ionization mode.

Compound	Rt (min)	Precursor Ion (*m*/*z*)	HPLC-ESI-MS^n^ *m*/*z* (% of Base Peak)	Compound Identity	Refs
1	1.26	387	MS2 [387]: 341(100) MS3 [341]: 179 (100), 119 (70), 113 (50), 161 (45), 143 (40), 131 (20)	1-Caffeoyl-β-D-glucose	[30]
2	2.96	133	MS2 [133]: 115 (100), 71 (10)	Levulinic acid	[44]
3	3.24	191	MS2 [191]: 173 (100), 111 (50), 155 (10)	Quinic acid	[32]
4	10.38	151	MS2 [151]: 136 (100), 123 (5), 107 (5), 88 (5)	Vanillin	[31]
5	15.01	163	MS2 [163]: 11 9(100)	*p*-Coumaric acid	[31]
6	17.20	563	MS2 [563]: 443 (100), 473 (70), 353 (40), 383 (30)	Apigenin 8-C-arabinoside-6-C-glucoside	[33,34,35]
7	17.27	563	MS2 [563]: 473 (100), 443 (70), 353 (65), 383 (45)	Apigenin 6-C-arabinoside-8-C-glucoside	[33,34,35]
8	20.70	187	MS2 [187]: 125 (100), 169 (5), 97 (5)	Hydroxy gallic acid	[42,43]
9	22.46	355	MS2 [355]: 337 (100), 219(60), 325 (20), 204 (10), 176 (5)	Unidentified	-
10	33.16	329	MS2 [329]: 314 (100), 299 (5)	Tricin	[36]
11	35.80	329	MS2 [329]: 229 (100), 211 (90) 314 (70), 311 (20), 295 (30), 171 (10), 155 (40)	tri-OH oleic acid derivative	[46]
12	36.59	671	MS2 [671]: 329 (100), 314 (30)	Tricin derivative	[38,39]
13	38.32	315*	MS2 [315]: 271 (100), 269 (10), 199	2′,7 dihydroxy-4′,5′-dimethoxyisoflavone	[41]
14	42.14	311	MS2 [311]: 293 (100), 171 (50), 201 (40), 211 (30), 197 (35)	Unidentified	-
15	45.39	295	MS2 [295]: 171 (100), 277 (80), 179 (35), 195 (30)	Hydroxy-octadienoic acid	[45]

### 3.3. Evaluation of Antiglycative Capacities of Rice Husk Extract

**Figure 4:**
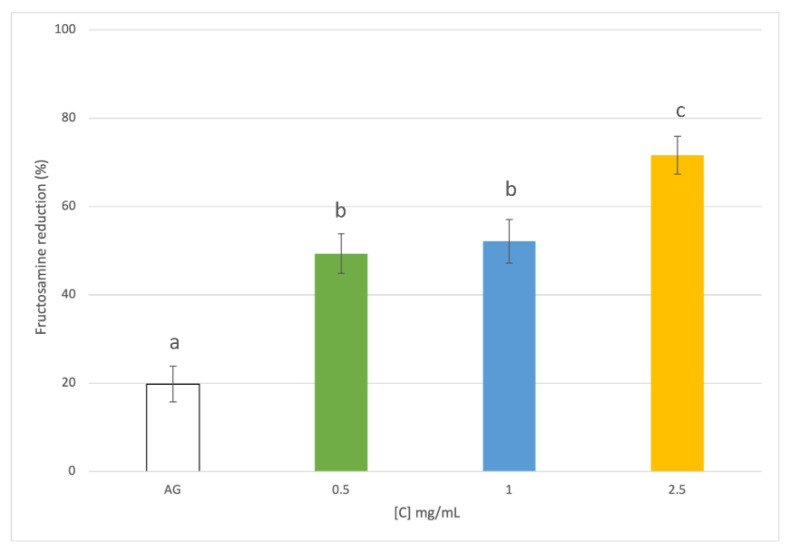
Inhibition of the fructosamine formation by rice husk extract tested at different concentrations (0.5, 1, and 2.5 mg dry matter/mL) and aminoguanidine (AG, reference standard) in NBT assay. Different superscript letters (a; b; c) indicate significant differences (*p* < 0.05) among the different concentrations of the tested extract and AG.

To the best of our knowledge, the potential activity of rice husk in the reduction of non-enzymatic protein glycation (the Maillard reaction) has never been investigated. Three different concentrations (0.5, 1, and 2.5 mg dry matter/mL) of RHE were investigated, monitoring the antiglycative properties at different stages of the non-enzymatic glycation of proteins. The effect of RHE on the early stage of the reaction was investigated by evaluating its capacity to inhibit fructosamine formation. Fructosamine is an Amadori product, generated by the rearrangement of Schiff’s bases, which originate from sugar carbonyl groups that react with protein amino groups after rearrangement, condensation, or oxidative modifications [47]. A BSA-GLU-based system was used to monitor the formation of fructosamine under mild conditions by NBT assay [18]. All the samples were able to reduce the formation of the Amadori products (Figure 4), with the lowest RHE concentration (0.5 mg/mL) reducing about 50% of the fructosamine. The activity increased with the increasing concentrationbut only when tested at 2.5 mg/mL (*p* < 0.05). Conversely, all the tested concentrations had a significantly higher activity than aminoguanidine (AG), which is generally used as a positive control due to its well-known action in the glycation process.

**Figure 5:**
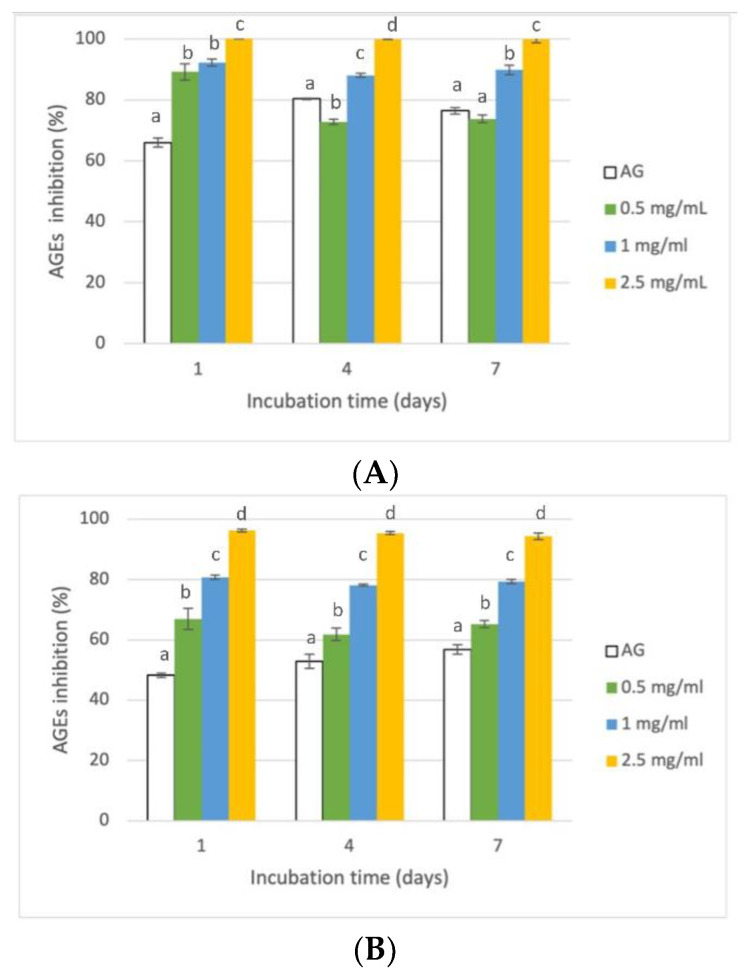
Inhibition of AGEs formation in BSA-MGO in vitro systems. (**A**) Inhibition of pentosidine-like AGEs formation (λ_ex_ 335 nm; λ_em_ 440 nm); (**B**) Inhibition of argpyrimidine-like AGEs formation (λ_ex_ 370 nm; λ_em_ 440 nm). Different superscript letters (a; b; c; d) indicate significant differences (*p* < 0.05) among the different concentrations of the tested extract and AG within each monitoring time.

Intermediate stages of the Maillard reaction are characterized by the generation of dicarbonyl compounds, which are considered advanced glycation end-products (AGEs) precursors [47]. The BSA-MGO system was set up to evaluate RHE action in the middle stage of glycation, as MGO can react with BSA, which acts as an intermediate for AGE formation. The capacity of RHE to act in this stage of protein glycation was studied by monitoring the pentosidine- and argpyrimidine-like AGEs formation in BSA-MGO systems, after 1, 4, and 7 days. A dose-dependent antiglycative activity was observed in both cases (Figure 5), and RHE was able to completely prevent the pentosidine-like AGEs formation during the entire monitoring period when tested at 2.5 mg/mL (Figure 5A), exceeding the activity registered for AG. Similar very high activity values (close to 100%) were registered for 2.5 mg/mL RHE when considering argpyrimidine-like AGEs (Figure 5B), but when tested at lower concentrations, RHE exhibited lower activity than the one registered for pentosidine-like AGEs.

**Figure 6:**
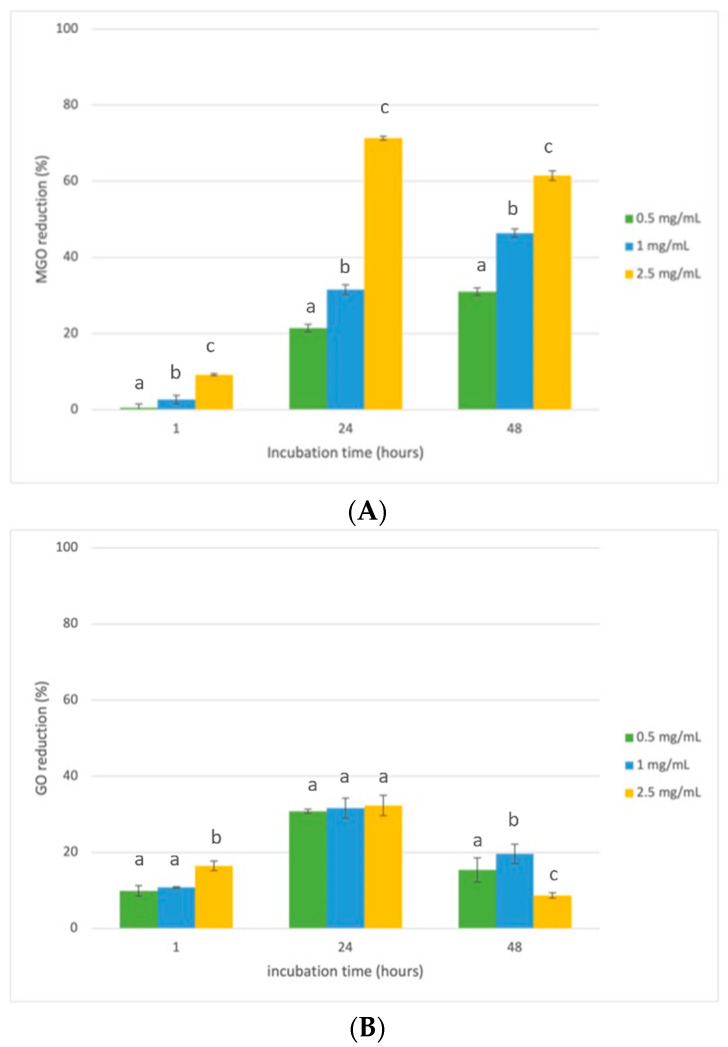
MGO (**A**) and GO (**B**) trapping ability of RHE at different concentrations (0.5, 1, and 2.5 mg/mL) after 1, 24, and 48 h of incubation. Different superscript letters (a, b, and c) indicate significant differences (*p* < 0.05) among the different concentrations of the tested extract within each monitoring time.

Polyphenols exhibit several antiglycation mechanisms, including the trapping of dicarbonyl species, thus reducing carbonyl stress [14,47]. Therefore, the RHE’s capacity to directly trap dicarbonyl compounds such as MGO and GO was evaluated in model systems at different times (1 h, 24 h, and h). RHE exhibited a promising MGO trapping ability (Figure 6A) but very low activity against GO (Figure 6B). This evidence was already reported in the literature, and it could probably be due to the easy polarization of GO in aqueous solutions, which is a limiting factor for its capture by quenchers [48,49].

**Figure 7:**
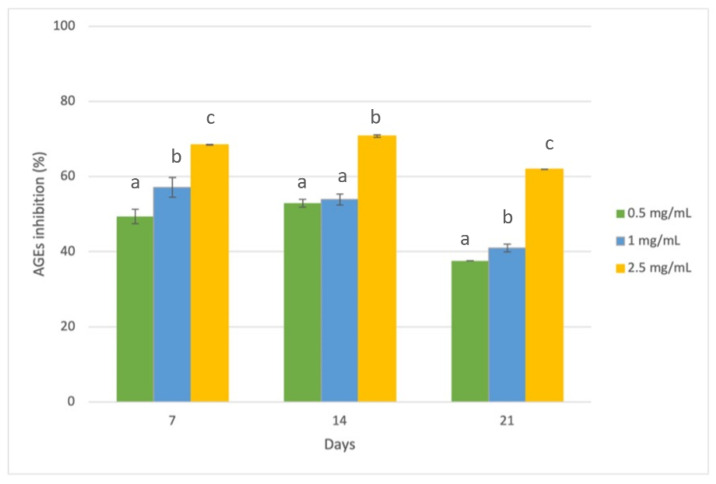
Inhibition of the AGEs formation in the BSA-GLU system by RHE at different concentrations (0.5, 1, and 2.5 mg/mL dry matter). Different superscript letters (a; b; c) indicate significant differences (*p* < 0.05) among the different concentrations of the tested extract within each monitoring time.

Considering the final stage of the glycation reaction, BSA-GLU systems were set up for monitoring argpyrimidine-like AGEs formation. Different monitoring times were selected considering the reactivity of glucose, including 7, 14, and 21 days (Figure 7) [50]. The results showed a promising dose-dependent RHE antiglycative activity, even if the differences between 0.5 and 1 mg/mL are not always significative (*p* < 0.05). The highest capacity to inhibit AGEs formation was recorded at 2.5 mg/mL after 14 days of incubation of the model system (70.8 ± 0.33%). After 14 days, a decrease in the glycated material’s fluorescence intensity was registered over the period of incubation. This behavior was related to the reaction kinetics of glucose, which tends to decline within 14 days, as already reported in the literature [18,19]. In general, AGEs formation is not always characterized by a linear trend, because it is the result of several chemical reactions in which intermediates form and rearrange [51].

**Figure 8:**
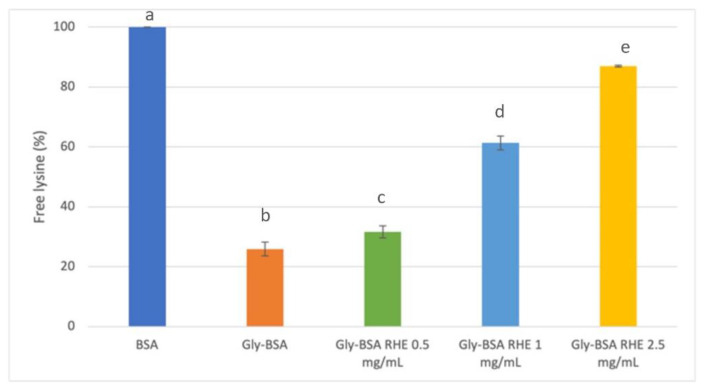
Free lysine levels in BSA-GLU and BSA-GLU-RHE (0.5, 1, and 2.5 mg RHE/mL) systems after 7 days of incubation at different concentrations. Different superscript letters (a; b; c; d; e) indicate significant differences (*p* < 0.05) in free lysine levels among the native bovine serum albumin (BSA), the glycated BSA systems (Gly-BSA), and the glycated BSA systems containing the different concentrations of the tested extract (Gly-BSA RHE 0.5 mg/mL; Gly-BSA RHE 1 mg/mL; and Gly-BSA RHE 2.5 mg/mL).

In order to gain more insight into the putative mechanism of polyphenols in inhibiting AGEs formation, the early progression of glycation reaction was evaluated by measuring the levels of free lysine in the BSA-GLU model after 7 days of incubation; in fact, this amino acid residue is involved at the beginning of glycoxidation process in the covalent bonding with reducing sugars [52]. Lysine residues in native BSA and glycated BSA (Gly-BSA) with and without RHE were estimated by OPA assay. Figure 8 showed that Gly-BSA exhibited a significant decrease in free lysine content in relation to the corresponding BSA value (*p* < 0.05). This decrease was indirectly correlated with RHE concentration. In fact, the highest free lysine content was registered for a 2.5 mg/ mL RHE (87.0±0.27%), followed by those registered for 1 mg/mL RHE (61.3%) and 0.5 mg/mL RHE (31.3%).

These results supported the hypothesis that lysine was involved in the glycation reaction and that RHE could be able to reduce its binding in a concentration-dependent manner.

Moreover, in order to investigate the potential correlation between antiglycative and antioxidant/antiradical activity, two different assays, i.e., the ABTS^•+^ cation radical scavenging activity and the DPPH free radical scavenging capacity, were set-up. The obtained results are reported in Table 5. RHE showed high capacity in scavenging ABTS^•+^ radical cations, especially when tested at 2.5 mg/mL (95.12%). Otherwise, only a weak capacity for reducing oxidative substances, such as DPPH radicals, was registered, and it appeared to be dose-dependent.

In addition, a strong positive correlation between antiglycative and antioxidant activity was found. As shown in Table 6, Pearson’s correlation coefficients (R^2^) were always higher than 0.9 for RHE between the two activities. Therefore, antioxidant compounds present in the extract might have a role in countering the glycation process, whose ability is directly related to their concentration. Effectively, the second step of the glycation reaction is characterized by the free-radical conversion of the Amadori products into AGEs, with the generation of reactive oxygen species (ROS) and reactive carbonyl species (RCS).

Several studies pointed out the good efficiency of cereal by-product extracts as antiglycative agents, such as those obtained from rice bran [53,54], purple corn cob [55], sorghum buckwheat hulls [56], and triticale bran [57]. The inhibitory effect on AGEs registered in the middle stage of glycation for RHE is similar to that reported for purple corn cob extract in BSA-MGO systems by Ferron et al. [55], characterized by a complete prevention of AGEs formation. Our promising RHE antiglycative capacity results are in good agreement with those presented by Rahman et al. [12], who reported glibenclamide-like antihyperglycemic activity of rice husk ethanolic extracts in glucose-loaded mice and supported their potential use to alleviate high glucose levels in diabetic patients. However, there are limited reports on rice husk antiglycative activity in vitro, and generally promising studies are focused on rice bran of colored varieties [54,58]. Our results are comparable with those obtained in BSA-GLU systems by Premakumara et al. [54] for red varieties of rice bran (80–90% of vesperlisine-like AGEs inhibition) and generally more promising than those obtained for white varieties, which inhibited 30% AGEs at the tested concentrations. The differences could be due to the polyphenolic composition of the extracts, which is generally correlated with the antiglycative activity. In particular, previous studies on rice milling by-products indicated that RHE has a better antioxidant phytocomplex than bran extract [4,59]. In addition, structure-activity studies indicated that C-glycosylated flavones and flavones are stronger AGEs inhibitors than flavonols, isoflavones, and flavanones [60,61]. RHE is rich in C-glycosilated apigenin, hydroxycinnamic and hydroxybenzoic acid derivatives, and flavonols, a composition that could support the registered antiglycative and antioxidant activity.

## 4. Conclusions

In this work, a polyphenolic extract obtained from organic rice husk was investigated for food purposes as an antiglycative and antioxidant ingredient. Following the need to explore innovative green extraction methods, a MAE coupled with hydro-alcoholic solvents was set up and optimized to recover polyphenols from husk with a DOE approach. The influence of temperature (T = 40–80 °C), percentage of ethanol (EtOH = 30–70%), time (5–35 min), and solvent to solid ratio (SSR = 15–35 g/mL) were initially studied using a Full Factorial Design to identify the significant factors affecting the quality process. Temperature and percentage of ethanol in the hydroalcoholic mixtures resulted in a positive effect on the recovery of polyphenols. Thus, only these two factors were further optimized (T = 60–90°C; EtOH = 60–80%) by using a Box Behnken design, maintaining the time fixed at 5 min and SSR at 1:35 mg/mL. Results showed that the optimal conditions were 80% EtOH, 90°C of T, 5 min of extraction time, and 1:35 g/mL of SSR. The richest in polyphenols extract thus obtained (RHE) was able to counteract the AGEs formation in all the assays used to reproduce the in vitro glycation reaction, which consisted mainly of incubation systems of a protein and a sugar, or MGO. In particular, RHE was active in inhibiting the fructosamine formation in the NBT assay (early stage of reaction) with a dose-dependent activity (50% of inhibition registered for the low concentration tested), showing values that were significantly higher than those obtained for AG. The antiglycative activity of the extract was more evident in BSA-MGO systems (middle stage of reaction), where a total inhibition of pentosidine-like AGEs at the highest tested concentration (2.5 mg/mL) was observed. Moreover, RHE showed good trapping abilities against MGO and pointed out a possible mechanism of action that needs further study to be explained. Considering the extract’s anti-radical capacity, a strong positive correlation was also found with its antiglycative activity (Pearson’s coefficient was always higher than 0.9), highlighting that the antioxidant compounds present in the extract were also involved in inhibiting AGEs formation. RP-HPLC-DAD-ESI-MS^n^ confirmed the presence of different polyphenols, mainly hydroxycinnamic acids and flavonoids, whose antioxidant and antiglycative abilities were already reported in the literature. The research is going on to investigate the extract components’ bioaccessibility and stability in order to obtain a rice-husk-based ingredient for food applications.

## Figures and Tables

**Table 1:** Full factorial design matrix and obtained response values, expressed as the total area of the peaks recorded in RP-HPLC-DAD chromatograms at 320 nm.

Sample	Ethanol (%)	Temperature (°C)	Time (min)	Solvent to Solid Ratio (mL/g)	Yield (mAU*s)
1	30	40	5	15	44,026.1
2	30	40	5	35	53,250.2
3	30	80	5	15	73,121.4
4	30	80	35	35	67,167.3
5	30	40	35	15	46,771.2
6	30	40	35	35	60,918.1
7	30	80	35	15	10,712.0
8	30	80	5	35	69,380.9
9	70	40	5	15	62,104.9
10	70	40	5	35	79,735.8
11	70	80	5	15	93,851.4
12	70	80	5	35	108,931.0
13	70	40	35	15	71,067.7
14	70	40	35	35	84,906.3
15	70	80	35	15	111,471.3
16	70	80	35	35	128,500.3
17	50	60	20	25	94,467.9
18	50	60	20	25	97,625.2
19	50	60	20	25	98,631.8

**Table 2:** Box-Behnken design matrix and response values obtained, expressed as the total area of the peaks recorded in RP-HPLC-DAD chromatograms at 320 nm. The actual yield refers to the obtained experimental data; the predicted yield is the value predicted by the applied statistical model.

Sample	Ethanol (%)	Temperature (°C)	Actual Yield (mAU*s)	Predicted Yield (mAU*s)
1	60	60	102564.9	102,381.0
2	80	60	96,972.2	99,914.4
3	60	90	120,588.4	118,411.0
4	80	90	136,819.6	137,771.0
5	60	75	98,178.2	100,539.0
6	80	75	112,884.1	108,987.0
7	70	60	90,576.6	87,815.2
8	70	90	113,531.8	114,757.0
9	70	75	89,423.5	91,429.1
10	70	75	91,898.6	91,429.1

**Table 3:** The analysis of variance of the Box-Behnken model for the phenolic compounds MAE yield.

Source	Sum of Squares	Df	Mean Square	F Ratio	*p* Value
A:EtOH	1.07056 × 10^8^	1	1.07056 × 10^8^	8.83	0.0411
B:T	1.08881 × 10^9^	1	1.08881 × 10^9^	89.83	0.0007
AA	4.14862 × 10^8^	1	4.14862 × 10^8^	34.23	0.0043
AB	1.19071 × 10^8^	1	1.19071 × 10^8^	9.82	0.0350
BB	2.26714 × 10^8^	1	2.26714 × 10^8^	18.70	0.0124
Total error	4.84828 × 10^7^	4	1.21208 × 10^7^		
PRESS	4.92509 × 10^8^				
R^2^	0.9772				
R^2^_adj_	0.9487				

Df: degree of freedom. _adj_: coefficient of determination.

**Table 5:** RHE radical scavenging capacity.

RHE Concentration	ABTS^•^^+^ Inhibition (%)	DPPH Inhibition (%)
0.5 mg/mL	41.55 ± 0.30	20.89 ± 0.28
1 mg/mL	65.57 ± 2.28	31.59 ± 0.21
2.5 mg/mL	95.12 ± 2.06	47.49 ± 0.06

**Table 6:** Pearson’s correlation coefficients (R^2^) between antiglycative activities monitored at the end of the monitoring time in the different assays and RHE antioxidant activities at the different tested concentrations.

Assay	DPPH (R^2^)	ABTS^•+^ (R^2^)
NBT	0.9566	0.9399
MGO	0.9705	0.9818
GLU	0.9604	0.9443

## Data Availability

Data is contained within the article.
